# A self-determination theory and motivational interviewing intervention to decrease racial/ethnic disparities in physical activity: rationale and design

**DOI:** 10.1186/s12889-016-3413-2

**Published:** 2016-08-11

**Authors:** Lauren S. Miller, Richard H. Gramzow

**Affiliations:** 1 Syracuse University, 513 Huntington Hall, Syracuse, NY 13244 USA; 2 Syracuse University, 503 Huntington Hall, Syracuse, NY 13244 USA

**Keywords:** Self-determination theory, Motivational interviewing, Exercise, Physical activity

## Abstract

**Background:**

Although the mental and physical benefits of physical activity are well-established, there is a racial/ethnic disparity in activity such that minorities are much less likely to engage in physical activity than are White individuals. Research suggests that a lack of motivation may be an important barrier to physical activity for racial/ethnic minorities. Therefore, interventions that increase participants’ motivation may be especially useful in promoting physical activity within these groups. Physical activity interventions that utilized the clinical technique of motivational interviewing (MI) in conjunction with the theoretical background of self-determination theory (SDT) have been effective in increasing White individuals’ physical activity. Nevertheless, it remains unclear the extent to which these results apply to minority populations.

**Methods/Design:**

The current study involves conducting a 12-week physical activity intervention based on SDT and MI to promote physical activity in a racially/ethnically-diverse sample. It is hypothesized that this intervention will successfully increase physical activity in participants. Specifically, it is expected that minorities will experience a greater relative increase in physical activity than Whites within the intervention group because minorities are expected to have lower baseline levels of activity.

**Discussion:**

Results from this study will give us a greater understanding of the generalizability of SDT interventions designed to improve motivation for physical activity and level of physical activity.

**Trial registration:**

Clinical Trials Gov. Identifier NCT02250950 Registered 24 September 2014.

## Background

The mental and physical benefits of engaging in physical activity are well-documented. Research suggests that regular physical activity decreases individuals’ risk of depression and anxiety, cardiovascular disease, diabetes, multiple types of cancer, and reduces the likelihood that individuals will die prematurely [1–3]. Clearly, encouraging people to engage in physical activity is a major priority within the field of Public Health.

According to the Centers for Disease Control and Prevention (CDC), it is important to increase the proportion of adults who engage in moderate intensity aerobic activity for at least 150 min/week, vigorous intensity for 75 min/week, or an equivalent combination [4]. In addition to aerobic activity, the CDC recommends that adults engage in muscle-strengthening exercises on two or more days a week in order to work all major muscle groups, such as legs, hips, back, abdomen, chest, shoulders, and arms [4].

### Physical activity in minority groups

Racial/ethnic minorities are especially unlikely to engage in physical activity and tend to have poorer health outcomes. In a study with over 11,000 participants, researchers found that non-White participants were significantly less likely to engage in ideal levels of physical activity (i.e., at least 150 min of physical activity per week) than were White participants [5]. Minorities also are at an increased risk for developing obesity, heart disease, and stroke - diseases that are influenced by physical inactivity [6, 7].

There are many possible reasons why minorities engage in less physical activity. Specifically, environmental barriers to physical activity, such as not having access to gyms or parks and the belief that one’s neighborhood is not safe, have been widely reported [8–10]. Social and cultural factors, such as the lack of support from family and friends, and the perception that African Americans have more physically demanding work and less free time than other groups, also impact minorities’ likelihood of exercising [11]. Additionally, research suggests that psychological factors, such as lack of motivation, enjoyment, and self-efficacy, are especially important in understanding why minorities are less likely to engage in physical activity [12–14]. These environmental, social, and cultural barriers to physical activity contribute to minorities’ lack of motivation for physical activity [15]. However, rather than attempting to modify the social, cultural, and environmental factors that thwart participants’ motivation engage in physical activity, we seek to directly manipulate motivation. The exercise instructors in the current intervention will utilize motivational theories and clinical techniques in order to promote participants’ motivation to engage in physical activity.

### SDT & MI interventions

Because minority groups report less motivation to engage in physical activity, interventions that increase participants’ motivation may be essential in order to promote physical activity within these populations. Self-determination theory (SDT) is one theory that can be used to understand factors that drive motivation. According to SDT, humans are driven by three innate psychological needs: autonomy, competence, and relatedness [16]. Autonomy is defined as “experiencing a sense of choice, willingness, and volition as one behaves” [17]. Competence implies that one is able to affect the environment and to attain desired outcomes within it [18]. Lastly, relatedness refers to the desire to feel connected to others [19].

Self-determination theory states that the extent to which people are able to fulfill these three basic psychological needs has a profound impact on their mental and physical health outcomes. People who report greater autonomy, competency, and relatedness (i.e., who have high need fulfillment) tend to experience more positive mental health outcomes, as well as more positive physical health outcomes [20–23]. Given that individuals’ trait levels of self-determination can predict their mental and physical health, researchers have tried to enhance participants’ autonomous motivation within interventions to promote positive health behaviors or to prevent negative health behaviors.

Physical activity is one important health behavior that health researchers have tried to promote using interventions based on SDT. Although research shows that people acknowledge the many health benefits of physical activity, motivation to engage in physical activity may decline if individuals’ physical activity environment does not support their autonomy, competence, and relatedness [24–26]. Research suggests, however, that interventions derived from SDT can be used to re-establish participants’ intrinsic motivation by providing a need-supportive environment. After SDT physical activity interventions participants report that physical activity is interesting, challenging and enjoyable and that physical activity produced an increase in participants’ self-reported happiness and vitality [27, 28]. Given that engaging in physical activity results in positive emotions and attitudes, physical activity interventions should produce greater intrinsic motivation than interventions that target less intrinsically-motivated health behaviors (e.g., medical testing, dietary control, dental hygiene, etc.).

Additionally, the therapeutic approach of motivational interviewing (MI) has been used in conjunction with SDT. Motivational interviewing is a client-centered counseling style for eliciting behavior change by encouraging clients to explore and resolve ambivalence [29]. The four general principles of MI involve expressing empathy, developing discrepancy, “rolling with resistance,” and supporting self-efficacy [30]. First, the MI-adherent counselor must express empathy towards the client because people are more engaged when they feel accepted and valued. Second, when individuals experience discrepancy between their current behavior (e.g., leading a sedentary life) and their personal core values or life goals (e.g., their desire to be physically healthy), this motivates individuals to align their behaviors with their values and goals. Third, MI states that the therapist should encourage participants to explore their ambivalence, rather than argue for change, which could actually make participants resistant to change. Fourth, supporting self-efficacy is important in MI because participants are more likely to try to change their behavior if they believe that they have the resources to overcome barriers and to achieve desired outcomes. These four principles of MI encourage people to engage in “change talk” – to verbalize their ability, desire, need, and reasons to change their current behavioral patterns. This change talk increases participants’ commitment to change, which predicts actual behavior change [29].

Many researchers have commented on the ways in which the combined effect of these two theories could be used to elicit greater behavior change [31–36]. Specifically, SDT may be used to explain how and why MI interventions work by providing psychological mediators that explain MI intervention efficacy [34]. SDT interventions can also be used to create need-supportive environments which bolster participants’ motivation to engage in desired behaviors [37]. Additionally, MI can provide SDT researchers with a concrete set of methods (e.g., reflective listening and open-ended questioning), which have been shown to increase participants’ motivation to change their behavior [36]. As argued by Vansteenkiste and Sheldon [36], combining the applied approach of MI and the theoretical approach of SDT should be beneficial to the progress of both motivational perspectives. Many research articles have discussed the theoretical importance of using SDT and MI within interventions [31–36]. Other research has demonstrated that SDT and MI interventions impact participants’ physical activity [38–41].

Although there is evidence that interventions based on both SDT and MI are effective in promoting greater physical activity, this has been studied among primarily White samples and there is a dearth of information on their effect on physical activity within minority groups [38, 40, 41]. There is, however, evidence for the efficacy of interventions based on SDT and MI, when used separately within minority populations. The previous research has found that MI produces greater behavioral change within domains such as alcohol use, smoking, drugs, HIV, treatment engagement, diet, physical activity, eating disorders, and gambling among minorities (e.g., African Americans) than Whites [42]. Due to the greater efficacy of MI within minority populations, it has been proposed that MI may be especially effective for individuals who are resistant or less ready for change [43]. There is also empirical and theoretical support for the efficacy of self-determination theory interventions on physical activity in minority populations. Previous intervention studies have shown that SDT interventions are effective in promoting physical activity among minorities [44–46]. In terms of theoretical support, the degree to which a culture is supportive can impact individuals’ need fulfillment [16]. Cultural norms in minority groups dictate that health promotion behaviors, such as engaging in physical activity, are viewed as White middle-class behaviors; whereas unhealthy behaviors, such as not engaging in physical activity, are viewed as in-group defining [47]. Therefore, there may be less support for physical activity within minority communities, causing minority individuals to experience less need fulfillment within this domain. Interventions based on SDT are designed to increase need fulfillment within the desired domain. Hence, it is reasonable to expect that a physical activity intervention based on SDT would facilitate greater need fulfillment for activity in minorities. Despite the evidence that separate interventions based on either MI or SDT are effective in helping participants to increase their physical activity, the combined efficacy of interventions based on both SDT and MI on physical activity within minority groups has yet to be examined.

Given that interventions based either on the theoretical perspective of SDT or the clinical perspective of MI have been shown separately to be effective in promoting health behaviors in minority populations, and that interventions combining techniques from these clinical and theoretical backgrounds have been shown to be especially effective in promoting physical activity (in largely White samples), it stands to reason that a physical activity intervention combining SDT and MI techniques would be the best option to motivate minority groups to engage in physical activity. The current study will utilize a physical activity intervention based on SDT and MI to examine the following hypotheses.

### Hypotheses for the primary outcome: physical activity

- *Hypothesis 1A*: White participants will report engaging in more physical activity than participants from minority groups at baseline (i.e., a significant main effect of race/ethnicity).
- *Hypothesis 1B*: There will be a greater increase in physical activity from baseline among the intervention groups than among the control groups (i.e., a significant main effect of condition).
- *Hypothesis 1C*: There will be a greater increase in physical activity from baseline for minority participants in the intervention groups than White participants in the intervention groups (i.e., a significant race/ethnicity by condition interaction).
- *Hypothesis 1D*: Participants in the intervention groups will experience a greater increase in need fulfillment during exercise (i.e., autonomy, competence, relatedness and intrinsic motivation) which will mediate the effect on physical activity.
  $$ Condition\to Need\  Fulfillment\to Physical\  Activity $$
- *Hypothesis 1E*: Participants in the intervention condition will report that their instructor utilizes MI skills more frequently which will mediate the effect on physical activity.
  $$ Condition\to Instructor\hbox{'}s\  Perceived\  use\  of\ MI\  Skills\to Physical\  Activity $$

### Hypotheses for secondary outcomes: need fulfillment, well-being, adherence

*Hypothesis 2A*. Participants in the intervention groups will experience a greater increase in need fulfillment, psychological well-being, and physical activity adherence than participants in the control groups (i.e., a significant main effect of condition).

## Methods/Design

The study has been approved by Syracuse University Institutional Review Board. The intervention is based on one motivational theory, SDT, and a clinical approach, MI, as well as fitness recommendations from the CDC (i.e., 150 min/week of moderate intensity or 75 min/week of vigorous intensity aerobic activity and muscle-strengthening exercises). The study will be conducted at two Syracuse-based YMCA facilities (i.e., the Downtown and Southwest YMCAs). These facilities were chosen because there is variability in the racial/ethnic composition of the clients in these locations. Due to the racial/ethnic composition of the staff at these YMCAs, all of the exercise instructors in this study will be White.

### Participants

An a priori ANOVA power analysis was conducted to determine how large a sample would be needed to detect significant main effects and interactions (G*Power version 3.1.3., 2010). This was estimated using a large effect size (f = 0.50), an alpha level of 0.05, and the power was estimated at 0.80. This power analysis determined that 48 participants are needed in the sample in order to detect a statistically significant change in an effect of this size.

#### Participants recruited

Participants were recruited using online advertisements (YMCA Facebook website) and hard-copy advertisements that were displayed at the YMCA and throughout the city of Syracuse at public libraries, community centers, churches, etc. If the YMCA members were interested in participating, they completed a survey online through Qualtrics or a hard copy version of the survey in order to determine whether they were eligible to participate in the study. The inclusion criteria mandated that participants were currently healthy enough to exercise once a week, not be pregnant or planning to get pregnant within the next 3 months, were willing to attend an exercise class once a week for 12 weeks, were willing to complete questionnaires at baseline and 12 weeks, allowed the intervention staff to monitor their attendance at the YMCA for 6 months post intervention, and allowed the exercise instructor to create an audio recording of all of the intervention sessions.

#### Participants enrolled

**Fig. 1:**
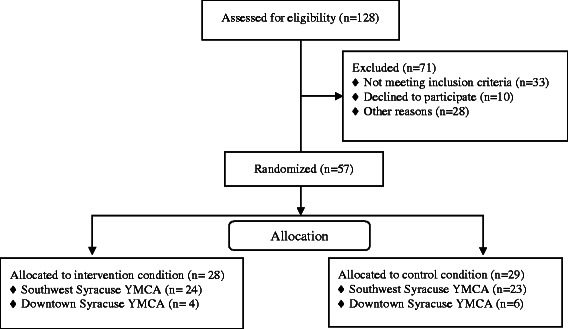
CONSORT flow diagram

Although 128 individuals completed the recruitment survey to enroll in the study, 33 did not meet the eligibility requirements, 10 did not attend any of the exercise classes, and 28 dropped out of the study because the YMCA director changed the time of an intervention class and control class at one site from an evening class to a morning class (see Fig. 1). In total, 57 participants enrolled, were randomized into the intervention or control condition, and attended the first exercise class. The experimenter obtained written informed consent from all participants before they participated in the study. Table 1 contains demographic information about the participants who attended this physical activity intervention. Participants’ age, education, racial/ethnic group, and sex did not significantly differ by condition (i.e., intervention or control).

**Table 1:** Characteristics of participants in the intervention, the control, and the total sample

Characteristics	Intervention (N = 27)	Control (N = 30)	Total (N = 57)
Age [M (SD)]	50.81 (11.63)	48.43 (11.99)	49.56 (11.78)
Females in sample	22 (81.5 %)	26 (86.7 %)	48 (84.2 %)
Race			
White	15 (55.6 %)	16 (53.3 %)	31 (54.4 %)
Black	9 (33.3 %)	11 (36.7 %)	20 (35.1 %)
Hispanic	3 (11.1 %)	1 (3.3 %)	4 (7 %)
Other	0	2 (6.7 %)	2 (3.5 %)
Education			
HS/Some college	5 (18.5 %)	5 (16.7 %)	10 (17.5 %)
Associates or Bachelors	7 (25.9 %)	15 (50 %)	22 (38.6 %)
Graduate Degree	15 (55.6 %)	10 (33.3 %)	25 (43.9 %)

*HS*/*Some college* High School graduate or attended some college but did not earn a degree

#### Assignment to condition

The current study is a parallel group, two arm, superiority trial with 1:1 allocation ratio. First, participants were allowed to choose whether they wanted to attend the Downtown YMCA or Southwest YMCA. Next, stratified randomization was employed such that participants were separated by race/ethnicity (i.e., minority versus non-minority) and participants were randomly assigned to the control or intervention condition within each site. A computer-generated allocation sequence was used and assignment to condition was made using sequentially numbered, opaque, sealed envelopes. Throughout the study, participants and the research assistant (i.e., an outcome assessor) will remain blind to condition. Due to the nature of the study, the exercise instructors and the experimenter (i.e., an outcome assessor and data analyst) will not be blind to condition.

### SDT and MI Intervention

**Fig. 2:**
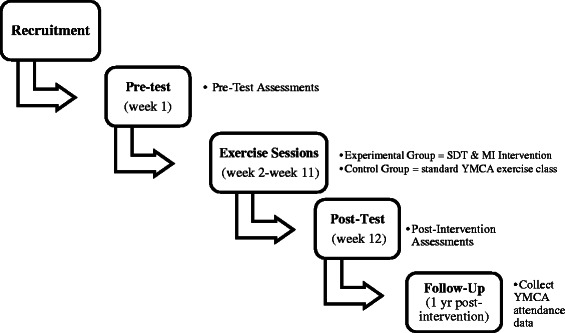
Timeline for the SDT and MI Intervention

All participants will participate in 12 weekly sessions for one hour with a YMCA instructor who has been trained to teach exercise classes. A brief intervention will be utilized because past research suggests that brief interventions based on both SDT and MI can increase participants’ physical activity and physical activity adherence over time [38, 41]. Participants in the intervention groups will engage in group discussions led by an SDT and MI-trained exercise instructor and will engage in exercise. The control groups will be taught by an exercise instructor who will not be trained in SDT and MI. The participants in the control groups will engage in exercise classes that are not based on any clinical or theoretical basis. This is an appropriate comparator since the control sessions closely resemble group exercise classes that are traditionally taught at gyms. Figure 2 shows a timeline for the intervention.

#### Intervention sessions

The MI techniques that will be used in the intervention sessions are primarily based on information from the books, Motivational Interviewing, Third Edition: Helping People Change and Motivational Interviewing in Groups [48, 49]. The Diabetes Prevention Program, a motivational program to increase physical activity and healthy eating, informs some of the curriculum that will be used during the intervention [50]. Additionally, articles by Williams et al. [51] and Edmunds, Ntoumanis, & Duda [37] inform the SDT techniques that will be used within the intervention. The content of the intervention sessions are described in Table 2. The exercise instructors have some flexibility during their classes in regards to what they decide to discuss within the class. There are two sessions (session 10 and 11), which are not designed to include specific material. Therefore, the instructors can decide to change the treatment protocol by discussing any issues that they believe would help the class during two sessions. They can decide to implement these sessions any time after the first session. The instructors’ standardized treatment manual will facilitate SDT- and MI-adherent conversations, but the program will be tailored to the needs of the group.

**Table 2:** Activities during the twelve intervention sessions

	Sessions											
	1	2	3	4	5	6	7	8	9	10	11	12
Aerobic and muscle-strengthening exercises	✓	✓	✓	✓	✓	✓	✓	✓	✓	✓	✓	✓
Pre and post-test survey	✓											✓
Worksheet: Music for class and how to use Facebook page	✓											
Worksheet and Class Discussion: Values, goals, and how PA fits in their lives		✓										
Worksheet: Types of PA they enjoy, Class Discussion: What they know about PA, more information about PA			✓									
Short IPAQ and CEMI				✓				✓				
Class Discussion: Feedback about the PA they do versus the CDC Guidelines				✓								
Worksheet and Class Discussion: If participant wants to change the amount or type of PA they do, confidence strengthening worksheet; if participant doesn’t want to change their PA, barriers to change worksheet.					✓							
Worksheet and Class Discussion: If participant want to change PA, signed contract describing their plan to change; if they don’t want to change PA, barriers to change worksheet.						✓						
Instructor has individual discussions with participants who do not have a plan to change their PA.							✓					
Worksheet and Class Discussion: Assess current level of progress with PA change. If unsuccessful, develop new plan.								✓				
Class Discussion: PA with family, friends and pets									✓			
Class Discussion: Instructor decides on topic and leads an SDT and MI-consistent discussion										✓	✓	
CEMI and Survey about participants’ perception of the study												✓

*Pre and post*-*test survey* The consent form, Demographics (age, sex, race, education, and income information), The Support for Exercise Habit Scale, Basic Psychological Needs in Exercise Scale, Behavioural Regulation in Exercise Questionnaire, The Integrated Regulation Items, and The Psychological General Well-Being Index, *PA* Physical Activity, *Short IPAQ and CEMI* Short version of the International Physical Activity Questionnaire and the Client Evaluation of Motivational Interviewing Scale

### Role and training of YMCA intervention staff

The exercise instructors who will implement the SDT and MI intervention will attend three standardized training sessions which will each last two hours long. The training and implementation of the intervention was developed based on the treatment fidelity framework, based on the Behaviour Change Consortium, to ensure the reliability within the intervention [52, 53]. Although, Miller and Rollnick [48] state that it takes approximately a decade of learning and actively using motivational interviewing to ensure proficiency, this type of training is not feasible at the YMCA. A literature review concludes that the amount of training that professionals receive in MI before conducting interventions varies greatly (i.e., less than 8 h of training to more than 24 h of training) and that the majority of these studies found positive outcomes relating to the development of MI knowledge, attitudes, basic skills, self-efficacy, interest in MI, and willingness to use MI [54]. Additionally, an intervention that examined the combined efficacy of SDT and MI on exercise and weight loss found a significant increase in exercise at the end of the 1 year intervention and 1 year post-intervention [40, 55]. This intervention used a two-day workshop in order to train their intervention staff in MI (M. Silva, personal communication, March 5, 2014). Therefore, it is expected that the current study will produce similar results.

The training sessions will teach the instructors about SDT and MI, as well as describe why this theory and clinical technique is used within our intervention. The training sessions will be taught by a clinical psychology Ph.D. student who has used MI in clinical settings and received training in MI from a licensed clinical psychologist, and the experimenter, a social psychology Ph.D. student who read extensively about MI, watched therapists utilize MI techniques, and role-played using MI techniques with the other trainer.

#### MI training

The instructors will learn about the spirit of MI (i.e., encouraging a partnership, providing acceptance, compassion and evoke individuals’ motivation to change), the four general principles of MI (i.e., express empathy, develop discrepancy, avoid argumentation, roll with resistance, and support self-efficacy), and how to use MI microskills (i.e., asking open-ended questions, using affirmations, using reflections, and using summaries) [56]. Instructors will also use exercises from the Motivational Interviewing Training for New Trainers, role-play situations (initially playing the role of the student and then take the role of the instructor), review the treatment manual, and watch taped sessions that depict the use of MI (i.e., MI spirit and microskills) [48, 57].

#### SDT training

To promote participants’ autonomy, instructors will give meaningful rationale for the activities and exercises used in the intervention, directions will be presented in a non-pressuring manner (e.g., “you could” versus “you should”), instructors will tailor the class to the participants’ needs and abilities so that they will experience an optimal level of challenge, and will give students choice regarding the difficulty and types of exercise used within the class [37, 58]. In order to promote participants’ sense of competence, instructors will provide participants with clear instructions during the intervention, and they will provide positive feedback when participants enact the desired behaviors [37, 58]. To satisfy participants’ relatedness needs, instructors will encourage positive relationships within the intervention setting by perspective taking, by noting verbal and non-verbal cues that indicate how participants feel during the intervention, and by working within participants’ comfort zones in order to make the intervention an interesting and rewarding experience [37, 58].

Additionally, the experimenter will meet with the exercise instructors in the intervention groups four times for half an hour during the intervention to discuss their use of SDT and MI techniques and to have them engage in a few role play exercises to strengthen certain skills. To increase the amount of practice that the instructors have using SDT and MI in the current study, the intervention exercise instructors will be encouraged to utilize these techniques within their other exercise classes and within their daily life. The experimenter will not meet with the exercise instructors in the control group during the intervention.

### Treatment fidelity assessment

Treatment fidelity will be assessed in order to determine whether the intervention is implemented as intended and whether the intervention groups differ from the control groups in terms of their use of SDT and MI techniques. Therefore, an audio recording will be created for each exercise instructor before they are trained in SDT and MI. These recordings will be used to determine the percent of MI- and SDT-adherent components that the exercise instructors demonstrated at baseline. An audio recording will also be created for all of the intervention sessions, which will be coded every fourth week as the intervention progresses. The sessions will be coded by the experimenter (who will not be blind to condition) and a research assistant (who will be blind to condition). High inter-rater reliability will provide evidence of agreement in use of the coding technique by both coders. This information will be coded in order to allow the experimenter to assess treatment fidelity at the different sites. Deviations from SDT and MI techniques will be identified and reviewed with the exercise instructors. The Motivational Interviewing Treatment Integrity Code (MITI) version 3.1.1 [59] will be used to assess the fidelity of the motivational interviewing components of the intervention, as well as participants’ language use within the sessions. Exercise instructors’ use of MI will also be assessed by the Client Evaluation of Motivational Interviewing Scale [60]. A self-created measure based on Wilson, Griffin, Saunders, Kitzman-Ulrich, Meyers, & Mansard [61] will be used to assess the fidelity of the SDT components of the intervention.

### Measures

Participants will complete a number of surveys during pre-intervention, session 4, session 8, and session 12. All of the data will be collected, entered into the computer, and maintained by the experimenter. A description of the primary and secondary outcome measures are listed below, as well as the measures used to assess treatment fidelity.

### Primary outcome measures

#### Physical activity

Physical activity will be assessed using the YMCA’s records of how often the participant came to the control or intervention class (as a measure of persistence), how often the participant attends the YMCA during the intervention and 6 months following the intervention (as a measure of adherence post-intervention). Physical activity will also be assessed using two self-report measures: one question to assess muscle-strengthening exercise from the Behavioral Risk Factor Surveillance System Questionnaire [62], as well as using the long and short version of the International Physical Activity Questionnaire (IPAQ) [63]. The IPAQ weights the frequency of strenuous, moderate and low intensity physical activity by its metabolic equivalent, which quantifies the energy expenditure in each level of physical activity based on its intensity.

### Secondary outcome measures

#### Psychological well-being

The Psychological General Well-Being Index (PGWBI) will be used as an assessment of psychological health [64]. This 22-item scale assesses individuals’ anxiety (e.g., “Have you been bothered by nervousness or “your nerves” during the past month”), vitality (e.g. “How much energy, pep or vitality did you have or feel during the past month”), depressed mood (e.g., “Have you felt downhearted or blue during the past month), self-control (e.g., “I was emotionally stable and sure of myself during the past month), general health (e.g. “Have you been bothered by any illness, bodily disorder, aches or pains”) and positive well-being (e.g., I felt cheerful, and lighthearted during the past month”). The items are answered on a 6-point scale which ranges from 0 (*None of the time*) to 5 (*All of the time*).

#### Need fulfillment during exercise

The Basic Psychological Needs in Exercise Scale (BSNES) will be used to assess need fulfillment during exercise [65]. This measure is comprised of 11 items and responses are provided on a 5-point Likert scale ranging from 1 (I don’t agree at all) to 5 (I completely agree). This measure assesses participants’ sense of autonomy (e.g., “I feel that the way I exercise is a true expression of who I am”), competence (e.g., “I feel that exercise is an activity that I do very well”), and relatedness (e.g., “My relationships with the people I exercise with are very friendly”).

#### Intrinsic motivation for exercise

Individuals’ motivation for exercise will be assessed using the 15-item Behavioural Regulation in Exercise Questionnaire (BREQ) by Mullan, Markland & Ingledew [66]. This measure includes four subscales, which are external regulation (e.g., “I feel under pressure from my friends/family to exercise”), introjected regulation (e.g., “I feel guilty when I don’t exercise”), identified regulation (It’s important to me to exercise regularly”) and intrinsic regulation (e.g., “I exercise because it’s fun”) in regards to engage in physical activity behavior. The BREQ was selected over the BREQ-2 (a measure which includes four items assessing amotivation) due the fact that the experimenter expected participants to have some motivation to exercise due to the fact that they volunteered to attend a weekly exercise class; hence, it is expected that adding the amotivation subscale would be of little benefit. Although integrated regulation is not included in the BREQ, Wilson, Rodgers, Loitz, and Scime [67] created a 4-item subscale to assess integrated regulation (e.g., “I consider physical activity consistent with my values”) which is meant to be used in conjunction with the BREQ and will be utilized in the current study.

#### Social support for exercise

The Support for Exercise Habits Scale will be used to assess how much social support participants get from their family and friends in regards to their exercise behavior [68]. Participants complete the questionnaire using a five-point scale that ranges from 0 (*none*) to 5 (*very often*). This 30-item scale is comprised of two subscales: participation/involvement (e.g., “Exercised with me”) and rewards/punishments (e.g., “Got angry at me for exercising”).

### Treatment fidelity measures

#### MI techniques

Exercise instructors’ MI proficiency will be assessed at both the instructor-level and at the participant-level. The percentage of MI-adherent responses will be audio recorded using the Motivational Interviewing Treatment Integrity Code (MITI), version 3.1.1 [59]. The experimenter will code every fourth session to detect MI-adherent responses within the language that the exercise instructors use while interacting with participants. Instructors’ MI proficiency will also be assessed by the students in their classes. The Client Evaluation of Motivational Interviewing Scale [60] will be used to assess whether the participants perceive the exercise instructors to be using MI skills every fourth session. This sixteen item scale assesses two subscales: relational and technical factors associated with MI proficiency using a 4 point Likert scale which varies from 1 (never) to 4 (a great deal). An example of the relational factors includes the item, “The exercise instructor changed the topic when I became upset about changing my behavior.” A technical item is, “The exercise instructor helped you talk about changing your behavior.”

### Planned statistical analyses

The analysis plan involves a 2 (race/ethnicity) × 2 (condition: intervention vs. control) factorial ANCOVA with pretest scores entered as covariates to test Hypotheses 1A-1C involving physical activity (assessed by the International Physical Activity Questionnaire [63]). Analysis of variance will be used to test Hypothesis 2A involving need fulfillment (assessed by the Basic Psychological Needs in Exercise Scale [65] and the Behavioural Regulation in Exercise Questionnaire [66]), physical activity adherence (assessed by how often participants attend the YMCA six months post-intervention), and psychological well-being (assessed by the Psychological General Well-being Index [64]). When examining whether mediators account for the relationship between intervention condition and physical activity, the Preacher and Hayes [69] nonparametric bootstrap approach will be used (i.e., Hypothesis 1D using the Basic Psychological Needs in Exercise Scale [65] and the Behavioural Regulation in Exercise Questionnaire [66] and Hypothesis 1E assessed by the Client Evaluation of Motivational Interviewing Scale [60]).

## Discussion

Despite the evidence that interventions based on SDT and MI are effective in increasing White individuals’ physical activity, the combined efficacy of SDT and MI interventions on physical activity within minority groups has yet to be examined. The current study will determine whether SDT and MI-based interventions can be used to decrease the racial/ethnic disparity in physical activity.

This study has a number of strengths. A carefully designed, clinically- and theory-driven intervention will be used. Additionally, a rigorous treatment fidelity assessment will be conducted to determine the extent to which the exercise instructors followed the research protocol and utilized MI and SDT techniques during the intervention, the effect of the intervention exercise, and ways to improve the quality of future studies.

The current study also has limitations. The primary outcome in this study is level of physical activity, which will be assessed using a self-report measure (i.e., the IPAQ) rather than an objective measure of activity. This method was chosen because the IPAQ is a valid measure to assess individuals’ overall level of physical activity [63]. Future studies should utilize self-report measures, as well as objective measures of activity. Another study limitation stems from the fact that the racial/ethnic composition of the exercise instructors in this study does not match the racial/ethnic composition of the sample. Research suggests that, “A culturally centered intervention must consider the role of ethnic and racial similarities and differences in the client–therapist dyad. This dimension brings into focus the consideration of ethnic and race matching in the client–therapist dyad, as it may be important to acknowledge ethnic, racial, or cultural similarities and differences.” [70, 71]. Unfortunately, only White instructors are available to teach at the Southwest Syracuse YMCA and the Downtown Syracuse YMCA during the course of the intervention, and certain classes that are predominantly composed of minorities will not be taught by an instructor who is a racial/ethnic minority.

Given the Public Health concern posed by the fact that minorities are at an increased risk of becoming obese and developing chronic diseases associated with inactivity, this study will examine whether this type of intervention could cause a meaningful increase in motivation in this population, as well as a corresponding change in level of physical activity [72]. Additionally, this research should motivate future studies to examine whether interventions based on SDT and MI can motivate minority populations to engage in and maintain other positive health behaviors, such as utilizing preventative medicine or making healthier dietary choices.
